# Clinical Outcome Assessment Tools for Evaluating the Management of Gliomas

**DOI:** 10.3390/cancers17101659

**Published:** 2025-05-14

**Authors:** Sachiv Chakravarti, Sneha R. Gupta, Saket Myneni, Mazin Elshareif, James L. Rogers, Chad Caraway, A. Karim Ahmed, Karisa C. Schreck, David O. Kamson, Matthias Holdhoff, Victoria Croog, Kristin J. Redmond, Chetan Bettegowda, Debraj Mukherjee

**Affiliations:** 1 Department of Neurosurgery, Johns Hopkins University School of Medicine, Baltimore, MD 21205, USA; 2 Department of Neurology, Johns Hopkins University School of Medicine, Baltimore, MD 21205, USA; 3 Department of Neurosurgery, Vanderbilt University School of Medicine, Nashville, TN 37232, USA; 4 Department of Oncology, The Sidney Kimmel Comprehensive Cancer Center, Johns Hopkins University School of Medicine, Baltimore, MD 21205, USA; 5 Department of Radiation Oncology, The Sidney Kimmel Comprehensive Cancer Center, Johns Hopkins University School of Medicine, Baltimore, MD 21231, USA

**Keywords:** glioma, patient-reported outcome measures, quality of life

## Abstract

In recent times, a greater emphasis has been placed on framing patient clinical assessments and QOL as both endpoints and prognostic factors for neuro-oncological care. However, there is currently a lack of consensus on the most effective metrics and instruments for use in patients with primary brain tumors. Due to the heterogeneity in clinical characteristics, histological classification, anatomical location, and available treatment modalities for glioma, quantifying patient clinical condition and QOL within this unique population is particularly challenging. In this paper, we comprehensively review the available literature on the use of clinical assessment tools in glioma patients, highlighting the strengths and limitations of prominent instruments. We demonstrate that clinical outcome assessment (COA) instruments provide valuable and complementary insights into the physical, psychological, and spiritual well-being of glioma patients.

## Simple Summary

Brain tumor patients face unique challenges that affect their quality of life and overall well-being. Until now, doctors and researchers have struggled to agree on the best ways to measure and track these impacts. Our review examines the various assessment tools currently used for patients with gliomas—a common type of brain tumor. These tumors are particularly difficult to evaluate because they vary greatly in their characteristics, location, and available treatments. We looked at how different measurement tools capture physical abilities, psychological health, and spiritual well-being in these patients. Our findings show that comprehensive clinical assessments provide valuable insights that complement traditional medical outcomes. By better understanding how to measure quality of life and daily functioning in brain tumor patients, healthcare providers can deliver more personalized and effective care. This approach ultimately aims to improve treatment decisions and enhance overall patient well-being, ensuring that care addresses not just the disease itself but the whole person living with it.

## 1. Introduction

Despite accounting for less than 2% of all cancers, tumors of the central nervous system (CNS) are associated with a disproportionate risk of morbidity and mortality [1,2]. Stemming from glial cells in the CNS, gliomas are the most common primary brain tumors and present with significant heterogeneity in anatomical location, grade, and clinical features. Despite rigorous investigation into new therapeutic approaches and treatment options, outcomes for patients with gliomas, particularly high-grade gliomas, like glioblastoma (GBM), have remained poor. Indeed, glioma patients often report debilitating physical and psychological symptoms, which frequently persist through courses of aggressive multimodal therapy, including surgical resection, chemotherapy, and radiotherapy [3,4].

In recent times, a greater emphasis has been placed on improving clinical outcome assessments (COAs) for neuro-oncology cohorts, with a particular emphasis on developing a better understanding patient quality of life (QOL). Multifaceted in nature, QOL comprises several health-related domains, including emotional, physical, social, and spiritual well-being [5]. Notably, there has been more emphasis placed on these outcomes due to the findings that worsened QOL and lower functional status are significant predictors of survival and long-term outcomes [6,7,8]. However, obtaining accurate measurements of QOL for glioma patients is challenging due to a lack of consensus on the utility of established COA instruments among brain tumor patients. Indeed, available instruments vary widely based on outcome measurement, psychometric properties, and administration method. However, recent efforts seeking to develop and validate brain tumor-specific COA instruments have shown promise, with variable applications and statistical strengths.

The purpose of the present study is to provide a comprehensive overview of established COA tools for glioma patients, indexing established tools by their measurement methodology: Patient-reported outcome measures (PROs), clinician-reported outcome measures (ClinROs), or performance outcome measures (PerfOs). This manuscript may serve as a guide to clinicians regarding optimal QOL instrument selection while highlighting the potential need for novel instrument development that can be used to inform clinical care in this vulnerable patient population.

## 2. Search Strategy

Three authors (S.C., M.E., and J.L.R) queried the current and historical literature published from January 1982 to July 2023 pertaining to patient-reported QOL tools for glioma using the PubMed (Medline) database. Keywords used for the search include “quality of life”, “QOL”, “patient reported”, “glioma”, “high-grade glioma”, “low-grade glioma”, and “glioblastoma multiforme”. After performing the search, the three authors screened pertinent articles by title and abstract, defining tools as a measure of PROs, PerfOs, or ClinROs. Case reports, patient series, clinical trials, and systematic reviews were included to develop a comprehensive overview of the established literature. Cited references for each publication were also screened for studies relevant to the present review. One author (S.C.) used the scale for the quality assessment of narrative review articles (SANRA) to evaluate the quality of all cited studies [9].

## 3. Patient-Reported Outcomes (PROs)

PROs are standardized assessment instruments that directly capture information from patients regarding their symptoms, functional status, or health-related quality of life [10]. Notably, these measures provide insights into the subjective patient experience, providing a view into a lived reality that cannot be obtained through clinical or physiological parameters alone. In recent times, PROs have gained prominence as endpoints for both clinical care and claims of treatment effectiveness while simultaneously emerging as valuable tools that may be used to direct routine clinical practice and facilitate communication between patients and providers [11,12]. This has allowed for some comparison of the management options given the overlap of domains assessed, including executive function, activities of daily living, and mood between the instruments. However, there is still significant heterogeneity in the form and measured content of PRO tools used in the current clinical research and routine practice, with a lack of consensus on what tools are most effective for glioma patient populations in particular [10]. Table 1 provides an overview of COA instruments previously used in glioma populations that are measurements of PROs.

## 4. EuroQoL-5-Dimension (EQ-5D)

EQ-5D is a generalized measure of QOL that can be used for a variety of health conditions [21]. The questionnaire is comprised of a descriptive section and a visual analogue scale. The descriptive section comprises questions in five domains, including anxiety and depression, discomfort and pain, lack of mobility, inability to perform self-care activities, and inability to perform normal activities. The visual analogue sale measures health status, asking the respondent to determine whether they are at their worst or best imaginable health state. Responses to each domain can be quantified alone or as a global health score. Resulting scores can be gauged against index values from representative populations [21,22]. The EQ-5D survey is available both online and in print in over 150 languages. The survey is self-reported and generally takes 2–5 min to complete [23]. EQ-5D results may be summarized using a 5-digit code or a single numerical index value. The conversion from raw survey responses to index values is unique to the average responses of people from a patient’s county of residence with established survey baseline values sets published for over 25 countries and counting [23]. Sagberg et al. took EQ-5D index values from 164 patients who underwent surgery for resection of glioma with KPS serving as an anchor. The authors found that patients whose functional status improved over time did not have significant changes in EQ-5D 3L scores (*p* = 0.13), but patients who had adverse outcomes postoperatively had significant reductions in EQ-5D 3L scores (*p* < 0.001) [13]. Additionally, Jakola et al. analyzed a separate cohort of 88 glioma patients undergoing surgery, finding that EQ-5D scores significantly correlated with KPS (*p* < 0.001) [24]. While not widely adopted, EQ-5D has demonstrated some utility as a general QOL in small cohorts of glioma patients.

## 5. European Organization for the Research and Treatment of Cancer Quality of Life Questionnaire (EORTC QLQ-C30)

With increasing emphasis on the multifaceted nature of health, patient-reported QOL tools have increasingly been incorporated into comprehensive patient assessments. EORTC QLQ-C30 is one of the first multidimensional tools to achieve widespread use in clinical trials and has been established as an effective method of assessing QOL in patients with cancer [25,26]. EORTC QLQ-C30 was first published in 1993 for use in lung cancer patient, but it has since been validated for use in patients with breast, ovarian, head and neck, and brain cancers, including patients with glioma [27,28,29,30,31]. EORTC QLQ-C30 consists of a 30-item questionnaire composed of five functional scales, including physical, role, cognitive, emotional, and social scales. The instrument also includes questions in three symptom scales, including fatigue, pain, and nausea/vomiting, as well as one global quality of life scale. Over the past 30 years, amendments to the original EORTC QLQ-C30 have included single items or questions that assess financial burden and frequently experienced symptoms in cancer patients inclusive of dyspnea, appetite loss, sleep disturbance, constipation, and diarrhea [27]. The EORTC QLQ-C30 survey is self-reported and may be administered through computerized adaptive testing (CAT), which is an electronic version of the questionnaire that changes based on responses to previous questions, a paper examination or a mobile application. In applicable cases, a physician may also choose to read questions aloud if a patient has limitations relating to visual acuity, cognitive function, or reading ability. The EORTC QLQ-C30 assessment takes approximately 10–15 min if administered through CAT but may take less time if the patient uses the EORTC mobile application [32,33]. Notably, EORTC QLQ-C30 has been translated into more than 60 languages, with additional survey versions for dialects. Survey development for individual languages is decentralized but full examinations are available in their respective publications with several being validated among global brain tumor populations [34,35,36,37,38]. Using the EORTC QLQ-C30 scoring manual, qualitative answers to survey questions can be transformed into raw scores ranging from 0 to 100, with higher scores corresponding to higher levels of function.

Among glioma patients, Mauer et al. examined the correlation between baseline symptom presentation, health-related QOL (HRQOL) as measured by EORTC QLQ-C30, and survival in 490 newly diagnosed GBM patients. When controlling for clinical factors, the authors found that the cognitive functioning (*p* = 0.0001), global health status (*p* = 0.0055), and social functioning (*p* < 0.0001) domains of EORTC QLQ-C30 were independently and significantly associated with overall survival [14]. However, the authors note that the reliability and validity of classical multivariate analytical techniques may be diminished for HRQOL scales given the strong intercorrelation between individual subscales and presence of outliers. As a result, Mauer et al. assessed differences in Discrimination C-indexes and Nagelkerke’s *R*^2^, measures evaluating a model’s ability to correctly predict a patient’s relative chance of mortality compared to other members in a cohort and variation present in an outcome accounted for by a prognostic variable, respectively, in models incorporating baseline clinical characteristics alone and models including clinical characteristics and HRQOL. Notably, the authors found that there was no significant improvement in Discrimination C-indexes and Nagelkerke’s *R*^2^ upon the incorporation of HRQOLs through the EORTC QLQ-C30 survey indicating that, while individual subscales on the EORTC QLQ-C30 are independently correlated with patient survival outcomes, the incorporation of the scales in multivariate models failed to improve the baseline predictive ability of the models if they were developed with clinical factors alone [14]. A separate study by Dirven et al. sought to establish minimally clinically important difference (MCID) thresholds for each individual subscale in the EORTC QLQ-C30, anchoring subscale score changes to the outcomes each subscale was attempting to quantify. For example, improvements in the physical functioning scale were anchored to performance status while changes in the appetite loss scale were anchored to the number of anorexia events as defined by Common Terminology Criteria for Adverse Events (CTCAE). The authors found that MCIDs were in the range of 4–11 points, establishing a new standard for how such scores may be interpreted for ongoing and future glioma trials [39].

## 6. EORTC QLQ-BN20—Brain Cancer Module (BCM)

Developed for use in conjunction with QLQ-C30 and other generalized QOL assessments in clinical trials, EORTC QLQ-BN20, also known as the brain cancer module (BCM), consists of a 24-item scale with questions addressing five domains: future uncertainty, visual disorder, communication deficits, motor dysfunction, and emotional distress. Notably, the set of four questions associated with the emotional distress domain are based on the QLQ-C30 emotional distress domain and are commonly omitted when both scales are used in tandem. Furthermore, seven items of the questionnaire evaluate specific symptoms, often of particular concern to glioma patients, including headache severity, presence of seizures, prolonged drowsiness, and weakness of both legs [40]. EORTC QLQ-BN20 is available through a computerized adaptive assessment (CAT), on paper or a physician-facilitated online modality through the EORTC Quality of Life Organization website. Both the full QLQ-C30 and QLQ-BN20 surveys are available in over 40 languages and are in the process of being validated amongst global brain tumor cohorts. QLQ-C30 and QLQ-BN20 together generally take 15–20 min to complete but the QLQ-BN20 module alone may take less time. Individual subdomains for QLQ-BN20 are measured on a Likert scale in the range of 1–4 with a subsequent conversion to a linear 0–100 scoring range for a patient’s global QoL score [15,40,41,42].

Widely used internationally for assessing QOL in brain tumor patients, QLQ-BN20 was first described in a 1996 publication by Osoba et al., who found differences in QLQ-BN20 domain responses that reflected declines in neurological status as described in doctor–patient encounters [15,41]. Furthermore, the authors found that QLQ-BN20 motor dysfunction domain responses correlated with KPS and a modified version of the Barthel Activities of Daily Living Index (BADLI) [15,41]. In a 1997 follow-up study among 105 patients with newly diagnosed or recurrent glioma, the combination of QLQ-C30 and QLQ-BN20 was found to have sufficient internal consistency and test–retest reliability. Furthermore, the authors found that newly diagnosed patients with high KPS scores (80–100) had greater physical and cognitive functioning relative to those with diminished BCM subdomain scores or patients with recurrent glioma [42]. Importantly, a 2009 study by Bosma et al. analyzing 16 short-term-survival and 16 long-term-survival high-grade glioma patients found differing results from the earlier studies by Osoba et al. [15,41]. Indeed, Bosma et al. found that, among short-term high-grade glioma patients, only the leg weakness domain of QLQ-BN20 decreased significantly from the baseline to 4 months postoperatively (*p* = 0.026) [43]. Furthermore, among long-term survivors, the future uncertainty domain improved between the baseline and 8 months (*p* = 0.041), 12 months (*p* = 0.010), and 16 months (*p* = 0.002); the headache measure improved between the baseline and 12 months (*p* = 0.047); and the leg weakness measure improved between the baseline and 12 months (*p* = 0.046) [43]. These results demonstrate QLQ-BN20 may hold some prognostic utility for long-term high-grade glioma survivors [15,41,42,43].

## 7. Patient-Reported Outcome Measurement Information System (PROMIS)-10 and -29

The PROMIS instrument was launched by the National Institutes of Health (NIH) as a patient-reported QOL measure for general populations struggling with chronic diseases [44,45]. The PROMIS-10 and PROMIS-29 instruments serve as shortened versions of the full PROMIS questionnaire, consisting of 10 and 29 items or questions related to a patient’s physical functioning, ability to participate in social roles and activities, pain levels, sleep quality, fatigue, depression, and anxiety. Similar to EORTC QLQ-C30, the PROMIS survey is self-reported and may be administered as a computer adaptive test (CAT), short-form examination through an online health portal, such as REDCap, EPIC or other hospital application programming interfaces (APIs), a paper examination, or a mobile application. PROMIS examinations may also be read aloud to patients if a patient has limitations relating to visual acuity, cognitive function, or reading ability. Administration of the PROMIS questionnaire differs based on version, patient population, and chosen subdomains, but examinations generally take between 10 and 15 min for short-form assessments and 5 min for CATs and mobile application tests [44,45,46]. Notably, the PROMIS questionnaire has been translated into more than 60 languages with all translations available through the PROMIS health organization website. Each PROMIS item consists of a scale from 1 to 5 with increasing severity for higher numbers [47]. Raw scores from each measure are then converted to T-scores using a mean of 50 and a standard deviation of 10 for the US general population.

While validated for use in pre-operative surgical oncology patient populations and within the context of cancer rehabilitation, studies evaluating the utility of PROMIS in glioma patient populations are sparse. Romero et al. performed a pilot study integrating digital PROMIS questionnaires for brain tumor patients and their caregivers into existing nursing workflows and comparing PROMIS scores with EORTC questionnaire outcomes. Notably, for the 10 patients with malignant brain tumors included in the study, four of whom were diagnosed with GBM, the authors only found a significant correlation between the EORTC and PROMIS physical function domains (*p* = 0.04) [48]. Among glioma patients more broadly, Gabel et al. performed a retrospective review of 58 HGG and 21 LGG patients, evaluating functional impairment using the PROMIS and Neuro-QOL questionnaires. The authors found no significant difference in PROMIS scores between the HGG and LGG patient cohorts at diagnosis except for a correlation between the PROMIS pain intensity domain scores in LGG patients (*p* = 0.01) and lower PROMIS physical function scores in HGG patients (*p* = 0.05) [16].

## 8. Quality of Life in Neurological Disorders (Neuro-QOLs)

First published in 2012, the Neuro-QOL tool is a set of instruments commonly utilized to measure patient QOL relating to symptoms of neurological disorders. The tool consists of three individual domains (physical, mental, and social health), each of which include a set of 5–10 questions from 13 unique question banks, including inquiries about anxiety, depression, fatigue, activities of daily living, lower-extremity function–mobility, cognition–general concerns, cognition–executive function, emotional and behavioral control, overall well-being, sleep quality, ability to participate in social roles and activities, satisfaction with social roles, and sexual function and stigma [49]. Neuro-QOL short-form surveys are self-reported, with or without assistance from a caregiver, but are curated by physicians and administered through a computerized adaptive test (CAT) or paper examination. Neuro-QoL examination lengths are contingent on how many domains are included with each domain taking approximately 2 min to complete. Average examinations include 5–7 domains and require roughly 10–15 min to complete. The Neuro-QoL questionnaire is currently available in English and Spanish with translations in other languages currently works in progress. Raw scores from survey responses are commonly normalized to a mean score of 50 with a standard deviation of 10 for longitudinal comparisons of Neuro-QOL scores [49].

Despite widespread use across neurological disciplines, comparisons between the Neuro-QOL questionnaire and other validated QOL measures have yet to be described. However, Rogers et al. performed a descriptive study on patients with primary CNS tumors, collecting PROMIS, EQ-5D, MDASI-BT, and Neuro-QOL questionnaires from 248 long-term survivors of primary CNS tumors. The authors found that 19.8% of long-term survivors included in the study reported moderate-to-severe cognitive dysfunction as measured by the Neuro-QoL cognitive function short-form survey [4]. Gabel et al. performed a similar retrospective study among 58 HGG and LGG patients, finding that Neuro-QOL cognition scores were significantly correlated with aphasia severity in patients with both high- and low-grade gliomas (*p* = 0.0033) [16]. Given the relative paucity of studies describing the use of Neuro-QOL questionnaires in neuro-oncological populations, future studies are necessary to evaluate the specific utility of Neuro-QOL collective and short-form assessments in patients with brain tumors.

## 9. Thirty-Six-Item and Twelve-Item Short-Form Health Survey (SF-36, SF-12, and RAND-36)

The Medical Outcomes Study (MOS) Short-Form 36 was originally developed as a national longitudinal measure for generalized health outcomes and is composed of 36 single items organized into eight health concepts: inability to participate in physical activities, limitations in engagement with social activities due to physical or emotional problems, problems in fulfilling normal role activities due to loss of physical function, problems in fulfilling normal role activities due to emotional problems, pain, mental health, vitality, and general health perceptions [50]. SF-36 can be consolidated into physical-component and mental-component summary scores. SF-12 is a shortened version of SF-36, including the same subdomains as well as the same physical- and mental-component summary scores [51]. SF-36 and SF-12 examinations are self-reported and may be taken online or through a paper examination. Administration of the exam generally takes between 10 and 15 min for the full SF-36 but may take less for SF-12 [16,48]. The SF-36 and SF-12 surveys are available in English alone with unofficial translations in other languages being published by individual research groups. Raw responses to SF-36 and SF-12 can be converted into a linear 0 to 100 scale with higher scores suggesting better health status. Importantly, the SF-36 and SF-12 examinations are owned privately by Optum, Inc., and scoring formulas are not available publicly. However, the RAND-36 survey includes the same MOS survey items and, while scored differently, is available without additional cost [17,51,52].

Notably, the use of the SF-36 and SF-12 instruments in brain tumor populations is a relatively new development compared to other validated QOL tools. In 2017, Bunevicius et al. evaluated the reliability and validity of the SF-36 instrument among 227 brain tumors patients, of which 43 were diagnosed with a high-grade glioma. The author found that there was sufficient internal consistency among all SF-36 domains, except for the social functioning and general health subscales. Furthermore, patients with depressive symptoms, as measured by the Beck Depression Inventory-II (BDI-II), and those with low functional status, as measured by the Barthel Activities of Daily Living Index (BADLI), had lower scores across all SF-36 mental and physical domains, respectively [17]. Bosma et al. evaluated differences in SF-36 score trends between 16 long-term high-grade glioma and 16 short-term high-grade glioma patients, finding that long-term survivors reported fewer general health problems (*p* = 0.027) and had significantly improved physical-component summary scores (*p* = 0.034) at 4 months postoperatively compared to short-term survivors [43]. While only a handful of studies in glioma patients have been performed utilizing SF-36 questionnaires, these results indicate this instrument may be able to assist physicians with risk stratification within this patient population.

## 10. Linear Analogue Scale Assessment (LASA)

The LASA consists of five items quantifying a patient’s self-reported perception of their physical, spiritual, intellectual, emotional, and overall well-being. During the LASA survey’s original development, the examination was a visual analogue scale but, in its current rendition, is most used as a Likert scale where patients are asked to provide a rating from 0 to 10 for each domain. Higher scores suggest higher quality of life or perceived level of functioning. LASA surveys may be taken on paper or online, but physicians may also choose to facilitate survey administration if a patient experiences limitations related to cognitive function, reading ability, or visual acuity. LASA administration generally takes 5 min and the LASA has not been translated or validated in languages other than English [17,51,52]. Originating in psychological testing, the use of the LASA in oncological practice was first described by Priestman and Baum in 1976 but was popularized by Coates et al., who found a correlation between performance status and LASA scores for general well-being in patients with ovarian cancer, breast cancer, and melanoma [18,53,54]. Locke et al. performed a retrospective review of 205 patients with high-grade glioma, comparing differences in LASA scale responses to changes in the Sheehan Disability Scale (SDS), Profile of Moods (POMS) assessment, and Functional Assessment of Cancer Therapy-Brain (FACT-Br) tool over the course of chemotherapy and radiotherapy treatments [55]. The authors found the LASA scale was strongly associated with scores from the SDS, POMS, and FACT-Br assessments (*p* < 0.001) indicating that single-item LASA scales may serve as an alternative for all three established assessments. Brown et al. performed a similar analysis of the same dataset, presenting outcomes from self-administered LASA, SDS, POMS, and European Cooperative Oncology Group (ECOG) performance scores among 220 glioma patients. The authors found that lower LASA scores were correlated with worse ECOG performance scores (*p* < 0.001) [18,54]. Both Locke et al. and Brown et al. note the relative brevity of the LASA, pointing toward the potential utility of this tool in more frail or impaired patients for whom a shorter instrument may be desirable [55,56,57].

## 11. Functional Assessment of Cancer Therapy (FACT-G and FACT-Br)

The Functional Assessment of Cancer Therapy-General (FACT-G) is a 28-item questionnaire developed to assess physical well-bring, functional status, social interactivity, emotional state, and satisfaction with treatment in cancer patients. In addition to these 28 items, each of the five domains contains an experimental question that asks patients about the effect of each domain on their global QOL [58]. The FACT-G questionnaire is available for free in English through the FACIT organization. Examinations may be conducted online or on paper, and the exam may be self-administered or administered through a physician interview. Responses to the FACT-G can be converted into individual domain scores and a global score that are evaluated relative to distributions of scores from a specific patient sample.

The FACT-Br is a 50-item QOL questionnaire containing the 27 items from FACT-G along with 23 disease-specific questions related to brain neoplasms [56]. Similar to the FAC-G, the FACT-Br is available in online and paper modalities and may be self-administered or taken as an interview. The FACT-Br generally takes between 10 and 15 min and has been validated for use in a heterogenous group of brain tumor patients [58,59]. Translations of the FACT-Br may be acquired through registration from FACIT. FACT-Br translations undergo rigorous and centralized subject testing prior to becoming available to the researchers. Notably, FACT-Br has long been used in glioma randomized clinical trials as a comprehensive measure for patient QOL [19,60,61,62,63]. However, the utility of FACT-Br as a prognostic tool has been questioned in recent studies focusing on glioma and GBM. Peters et al. performed a prospective observational study of 237 patients with recurrent high-grade glioma finding that, in multivariable analysis, both the FACT-G and FACT-Br were not significant predictors of improved overall survival [64]. Furthermore, Roa et al. attempted to use the FACT-Br assessment to monitor QOL amongst 100 GBM patients >60 years of age, noting that only 45% of patients were able to complete the assessment. These results indicate that, due to assessment length and complexity, FACT assessments may be impractical to routinely administer in patients with decreased cognitive function [19,60,61,62,63,64].

## 12. MD Anderson Symptom Inventory-Brain Tumor Module (MDASI-BT)

The MDASI-BT was developed as a brain tumor-specific extension of the MDASI, a generalized measure of symptom severity for cancer patients. In addition to an assessment of 13 symptoms (pain, fatigue, nausea, disturbed sleep, distress, shortness of breath, difficulty remembering, lack of appetite, drowsiness, dry mouth, sadness, vomiting, and numbness and tingling) present in the MDASI questionnaire, the MDASI-BT asks about nine additional symptoms specifically related to neurological function, including unilateral body weakness; changes in appearance; changes in bowel pattern; vision problems; seizures; irritability; and difficulty with understanding, speaking, and concentrating [20,65,66]. Furthermore, the MDASI-BT assesses six “interference items”, including life enjoyment, work, mobility, activity, mood, and interpersonal relationships. Patients are asked to rate each symptom and interference item in the range of 0–10 with higher numbers indicating increasing severity of symptom presentation and greater interference in life activities. Patients may complete the MDASI-BT survey online or on paper. The survey can be administered through self-reporting or by interview from a healthcare professional. The full MDASI-BT survey generally takes 10 min. Full English and foreign language MDASI-BT exams are available for free through MD Anderson. Validation of the psychometric properties of foreign language MDAST-BT examinations are available through independent research groups collaborating with MD Anderson researchers.

Despite the relatively recent development of the MDASI-BT instrument, the evaluation of the tool’s utility has been well-described in the previous glioma literature. Acquaye et al. administered the MDASI-BT questionnaire and performed qualitative interviews with 23 patients with glioma, 11 of whom had GBM. The authors found that all symptoms reported in interviews were included in the MDASI-BT questionnaires with the exception of shortness of breath and dry mouth. These results validate that the MDASI-BT is broadly able to capture symptoms associated with a glioma diagnosis; respondents similarly noted symptom severity was also well accounted for in the questionnaire [67]. Building on these results, Vera et al. analyzed EQ-5D and MDASI-BT outcomes for 100 glioma patients, 78 of whom were diagnosed with GBM. The authors found that MDASI-BT, coupled with patient performance status, tumor grade, and recurrence status, provided greater granularity in patient assessment, describing 52% of the variability found in EQ-5D scores within this patient population [68]. These results indicate the MDASI-BT may serve as an easy-to-use instrument that provides a quick, but comprehensive, assessment of common symptoms affecting glioma patients. Use of the MDASI-BT in conjunction with other tools, such as KPS, may facilitate the evaluation of the relationship between cancer symptoms and disease trajectory for glioma.

## 13. Quality of Life Surrogates

In addition to these instruments, which were developed with the intention of assessing quality of life in patients, there has been growing evidence that symptom burden and functional status are effective proxies for quality of life. While not necessarily an exhaustive list, our search demonstrated that some of the following performance status and cognitive impairment tests have been found to be effective surrogates for QOL in glioma patients. Importantly, many of these scores are as predictive of tumor progression, disease recurrence, and overall survival as instrument derived for QOL evaluation.

## 14. Clinician-Reported Outcomes (ClinROs)

Clinician-reported outcomes (ClinROs) are standardized evaluation tools that require interpretation or judgement from a trained healthcare professional to assess a patient’s health status. Unlike patient-reported measures, ClinROs may capture aspects of diseases that are not directly reportable by patients, providing a structured quantification of clinical observations through rating scales or systems of classification [3,10]. Notably, the development and validation of ClinROs requires precise construct definitions, standardized measurement protocols, and a thorough evaluation of a tool’s psychometric properties. ClinROs play an important role in clinical trials and practice with applications spanning multiple domains of medicine. Table 2 provides an overview of COA instruments previously used in glioma populations that are measurements of PROs.

### 14.1. Karnofsky Performance Status (KPS) Scale

While not strictly measuring a patient’s QOL, the KPS scale is one of the most ubiquitous QOL-related tools found in the current medical practice. The KPS consists of an 11-point rating quantifying a patient’s ability to perform normal activities of daily living with scores range from 0 to 100 and increasing scores signifying greater functional status [71,72]. KPS measurements are subjective, assigned by physicians, and generally take 1–2 min [8]. Notably, KPS at diagnosis has been well established as a prognostic indicator for patients with glioma, although the metric has been criticized for failing to account for the complexity of neuro-oncological conditions. Indeed, Mackworth et al. analyzed 200 patients with primary brain tumors, finding that QOL was significantly correlated with freedom from depression (*p* < 0.0001), an active social life (*p* < 0.0001), fewer symptoms (*p* < 0.05), and high energy (*p* < 0.01), none of which are accounted for in the KPS scale. Furthermore, the authors found that KPS scores lost predictive significance when accounting for patient age and other clinical characteristics [69]. Given the lack of granularity associated with KPS scores, in recent times, this scale has largely been used as a measure of disability rather than QOL. Nevertheless, KPS remains widely used as an auxiliary measure of functional status, particularly for patients with impaired communicative ability and who may otherwise be unable to engage with more complex patient-centered QOL questionnaires [73].

### 14.2. Neurologic Assessment in Neuro-Oncology (NANO)

The Neurologic Assessment in Neuro-Oncology (NANO) is a tool that was developed by a consortium of multidisciplinary neuro-oncology providers aimed at assessing function across nine domains, including gait, strength, ataxia, sensation, visual fields, facial strength, language, level of consciousness, and behavior [74]. This instrument, which was constructed with the explicit goal of providing anatomically specific information regarding functional status and QOL, has been validated for a variety of brain tumors, including gliomas [74,75] The NANO has been integrated into several efforts to consolidate neuro-oncology patient-reported outcomes [10]. Despite its relative novelty, implementation in a few studies has shown that the tool has been an effective predictor of progression and survival that is as effective, if not superior, to general QOL and functional status metrics for glioma patients [70,76]. This has led to a growing movement toward implementing the NANO in recent guidelines and clinical protocols [77].

## 15. Performance Outcomes (PerfROs)

Performance outcomes (PerfOs) are standardized assessment measures that evaluate a patient’s ability to perform specific tasks under controlled conditions, yielding objective and quantifiable data about cognitive status or physical capabilities. PerfOs capture direct measurements of patient performance through heterogenous means, including time-to-completion, accuracy rates of quantitative scores based on an objective evaluation of functional domains [10]. Notably, PerfOs are valuable in therapeutic areas where changes in the capacity for a patient to perform specific tasks are an indicator for disease progression or treatment efficacy. This is particularly relevant for glioma patients as the progressive development of cognitive or sensory deficits may indicate disease recurrence, treatment side effects, or lack of a therapeutic’s function. Notably, PerfO development requires methodological consistency across divers patient populations and a careful standardization of scoring procedures in order to minimize measurement variability [1,2,3]. Table 3 provides an overview of COA instruments previously used in glioma populations that are measurements of PerfOs.

### 15.1. Montreal Cognitive Assessment (MoCA)

Similar to the MMSE, the MoCA serves as an effective screening tool for mild cognitive impairment and often can be a surrogate for QOL. MoCA is a 30-point rating that is attributed to patients based on an evaluation of several domains, including visuospatial processing, naming, concentration, language, abstraction, delayed recall, and orientation [78]. Completing the test takes about 10 min, and scores are assigned based on provider objective assessment of their patients’ performance [81]. Recently, providers have been using MoCA to assess prognoses, particularly postoperatively. For example, Tymowski et al. found that in a cohort of 21 patients, there was a significant decline in cognitive function immediately after surgery [81]. There have been several similar papers that have shown that the MoCA is a very helpful instrument for measuring cognitive impairment, a good proxy for QOL, in patients receiving surgery and adjuvant treatments for a glioma [82,83].

### 15.2. Trail Making Test (TMT)

The Trail Making Test (TMT) is a 5–10 min measure of cognitive function that utilizes a series of neuropsychological tests to provide a score that is interpreted based on an age-standardized curve [84]. This metric, which primarily assesses visual processing, fine motor skills, and executive function, has been shown to be a valuable instrument for assessing cognitive impairment over time [85]. Interestingly, TMT scores have been shown to directly correspond to glioma disease progression, with Wang et al. finding that older patients and patients with larger tumors had worse TMT scores [86]. Moreover, Smrdel et al. found that TMT provided the best discrimination between glioma patients with poor cognitive function and those with preserved function after implementing several well-known cognitive screening instruments [79]. As other studies have replicated these findings in various cohorts of glioma populations, it is clear that, while not directly measuring QOL, TMT is an effective measure of functional status and cognitive impairment in these patients and could be an alternative way to represent change in QOL over time [87].

### 15.3. Hopkins Verbal Learning Test (HVLT)

The Hopkins Verbal Learning Test or HVLT is an instrument that was developed primarily to assess working memory and word-finding functions [88]. While the HVLT was developed and implemented primarily as a predictor of dementia progression, recent studies have shown that it effectively measures symptom burden in glioma patients, especially those with tumors in complicated regions [80,89]. As a result, studies investigating the effectiveness of novel therapies, including modified radiation protocols and immunotherapies, have utilized HVLT scores as a proxy for assessing baseline and post-treatment symptom burden [90,91]. Notably, Valiulyte et al. found that changes in cognitive function scores assessed by the HVLT aligned with trends in QOL measured by validated QOL instruments in the glioma population [92].

### 15.4. Mini-Mental State Examination (MMSE)

In addition to loss of physical function, the importance of cognitive decline has been a well-documented surrogate for QOL in brain tumor-patient cohorts. The MMSE is an established screening tool for the generalized assessment of dementia and cognitive impairment, consisting of 11 questions that assess several areas of mentation, including attention and concentration, short-term memory, language skills, visuospatial abilities, and orientation to place and time [93,94]. A physician administrates the exam by asking and recording answers to a pre-determined set of questions. Furthermore, patients are required to write a short sentence and draw a picture of a displayed model as a part of the examination. A full MMSE generally takes 5–10 min but may take longer [93,94]. The full MMSE examination is available through the National Institutes of Health (NIH) and may be printed prior to exam administration. MMSE scores range from 0 to 30, with a score of 25 or higher generally classified as normal. Despite widespread use across general patient populations, the utility of the MMSE in glioma patient cohorts has been questioned. Indeed, the MMSE may not be sensitive in the upper range of scores with relatively poor sensitivity for mild cognitive impairment and some, but not severe or total, executive dysfunction [56,57,95,96]. Brown et al. evaluated changes in MMSE scores prior to and following radiotherapy treatment in 203 patients with low-grade gliomas and, despite considerable evidence to the contrary in the glioma literature, the authors found only a small portion of treated patients had cognitive deterioration as measured by the MMSE, indicating the need for a more granular neurocognitive assessment [56]. Nevertheless, Brown et al. also found that low-grade glioma patients with abnormal baseline MMSE scores (<26) in this cohort had significantly diminished 5-year progression-free (*p* < 0.001) and overall survival (*p* < 0.001) [57]. Taylor et al. similarly found patients who experienced a >3 point decrease in MMSE scores had a significantly shorter time to progression and death than their counterparts [92]. Furthermore, Gorlia et al. developed novel prognostic nomograms for overall survival in a cohort of 573 newly diagnosed GBM patients from a joint EORTC and National Cancer Institute of Canada (NCIC) trial, finding that MMSE scores >27 predicted improved median survival and probability of survival at 2 years [96]. These varied results demonstrate some utility for MMSE as a prognostic marker, while its overt use of a metric for QOL requires further study in glioma patients.

## 16. Discussion

Strategies to improve patient QOL have become increasingly important as new therapeutic approaches for brain tumor patients have been evaluated with the hope of extending survival. Though several QOL instruments are currently available, the field of neuro-oncology has been relatively limited in our approach to their routine use in patients with primary intracranial neoplasms.

### 16.1. Limitations to the Current COA Assessments

A proliferation of potential COA tools has occurred in conjunction with a renewed interest in studying QOL among glioma patient populations. Instruments used to measure clinical outcomes can be broadly categorized into tools that measure patient-reported outcomes (PROs), clinician-reported outcomes (ClinROs), and performance outcomes (PerfOs). Amongst all measures, the KPS, a ClinRO, appears to be one of the most widely used and well-established tools, with rapid assessment of disability level for glioma patients. While easily evaluated and standardized, the KPS is limited in its ability to capture the granularity of symptoms that impact patient QOL, especially amongst glioma patients who may experience complex, interlocking cognitive-, physical-, and mental-health related QOL deficits [5,6,7,8,9,10]. Similar concerns exist with the MMSE and LASA, ClinRO and PRO tools, respectively, with the sensitivity of both tools decreasing at the upper range of scores along with a very limited ability to capture nuances of executive dysfunction that can become significant barriers to QOL for glioma patients [13,22,23].

While having greater granularity and specificity for brain tumor populations, multi-faceted, neuro-oncology PROs may be more physically and mentally demanding, requiring additional time to complete [4,5]. Indeed, one of the largest barriers to the study of clinical outcomes in glioma patients is a lack of completed exams on a longitudinal scale and missing data, both of which may result in bias. Indeed, Renovanz et al. performed a retrospective QOL study using the EORTC QLQ-C30 and BN20 in a cohort of 173 glioma patients, finding that patients in a worse clinical condition and with a lack of motor function were more likely to drop out [97]. Similarly, Dirven et al. reviewed studies analyzing barriers to QOL assessment deployment in neuro-oncology practices, finding that administrative burden, a patient’s health status, and patient refusal were the most common reasons posed barriers to assessment compliance [98]. Furthermore, corroborated by Renovanz et al., Dirven et al. found that glioma patients who were compliant with QOL examinations had a better health status and improved long-term health outcomes [97,98].

Given these limitations, we propose a framework for the selection of PRO measures that can be utilized at varying stages of a patient’s journey following the diagnosis of a glioma.

### 16.2. A Framework for PRO Instrument Integration into Glioma Clinical Practice

**Figure 1:**
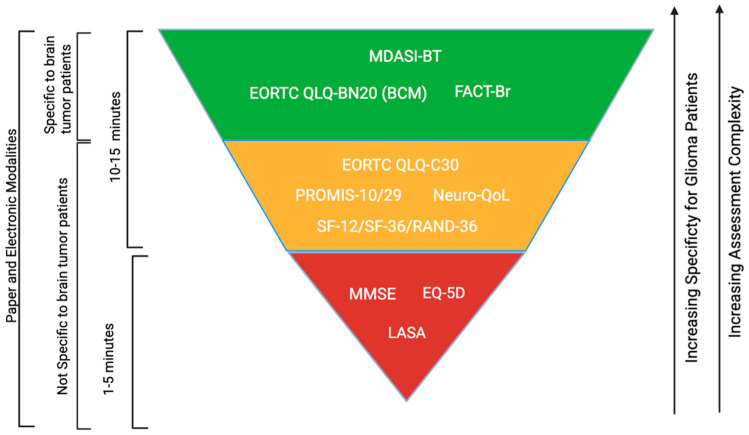
Graphical representation of characteristics for PRO instruments used in glioma patient populations.

Figure 1 provides a general graphical representation of characteristics for PRO instruments included in the present study. From red to green, instruments in a higher stratum have increasing specificity for glioma patients as well as assessment complexity. The MMSE, EQ-5D, and LASA were the shortest assessments, taking approximately 1–5 min, while the EORTC QLA-C30, PROMIS, Neuro-QoL, SF-12/SF-36, MDASI-BT, EORTC QLQ-BN20 (BCM), and FACT-Br have all been described to take between 10 and 15 min, with the potential for shorter examinations due to the modular nature of these instruments. Notably, all instruments include both an electronic and paper modality. 

In the green stratum, the MDASI-BT, FACT-Br, and Neuro-QOL capture the widest range of factors impacting brain tumor-patient quality of life with the greatest granularity. These tools take into account the development of broad symptoms that are related to neurologic dysfunction, including cognitive–executive function, fatigue, behavior, and motor function. Additionally, the MDASI-BT includes brain tumor-specific symptoms, such as seizures, vision issues, bowel and bladder dysfunction, language, and concentration concerns. Notably, the MDASI-BT, FACT-Br, and Neuro-QoL surveys all require high levels of engagement. As a result, these tools may be burdensome for patients who have cognitive, language, or attention deficits at the time of evaluation. In the yellow stratum, the EORTC QLQ-C30, PROMIS, Neuro-QoL, and SF-12/SF-36 assessments lack specificity for brain tumors but capture a wide range of general physical- and mental health-related QOL factors with high levels of granularity. For surveys in the green and yellow strata, the fatigue involved in the survey-taking process presents a significant challenge.

In the red stratum, the MMSE, EQ-5D, and LASA are all patient-reported QOL tools with moderate granularity and no specificity for brain tumor patients. Lack of granularity and specificity for brain tumor patients are the primary barriers for assessments in the red stratum. However, each survey takes 1–5 min and may be easily incorporated into the existing clinical workflows for glioma patients experiencing significant physical and cognitive deficits.

### 16.3. Sexual Health

Changes in sexual behavior may be the result of significant emotional distress from a brain tumor diagnosis, disruptions to interpersonal relationships, or the physical influence of the tumor itself [99]. Despite the importance of QOL disruptions associated with a loss of sexual function, scales evaluating changes in patient-reported QOL due to changes in sexual ability were largely omitted from the COAs described in the present review. Indeed, to our knowledge, the Neuro-QoL was the only instrument with dedicated items for sexual function [16,49]. There is a critical need to evaluate sexual function for glioma patients and develop instruments that provide standardized measures for sexual QOL [96].

### 16.4. Limitations

Our study has several important limitations. First, while we took steps to limit reviewer and selection bias, we may have failed to include studies investigating novel COA tools in glioma patient cohorts. Additionally, we were unable to incorporate studies or tools that were written in languages other than English. Regarding the works included in our review, a majority of the studies we describe were retrospective in nature and had small sample sizes. As a result, there may be methodological limitations to the use of the COA tools we describe that are inherent to the populations they were used to assessed. Several measures, such as the ECOG performance status and EORTC PRO measure, had few papers in the literature and as a result, we were unable to include substantive information about the measure in the present manuscript. Future studies should validate the tools we describe in diverse, heterogenous patient groups as well as in randomized clinical controls amongst glioma and glioblastoma patients.

## 17. Conclusions

With greater emphasis being placed on patient QOL, the development and rigorous evaluation of patient-centered COA instruments in brain tumor-patient populations are critical. Future research efforts are necessary to validate established instruments and improve upon both general and disease-specific COA tools for glioma patients.

## Figures and Tables

**Table 1:** Overview of patient-reported outcome (PRO) instruments used in glioma patients.

Instrument Name	Description	Number of Items	Domains	Time to Complete	Study Characterizing Use	Study Cohort
EQ-5D	EQ-5D is a generalized measure of QOL for patients with chronic diseases	5 individual items and 1 visual analogue scale	anxiety and depression, discomfort and pain, lack of mobility, inability to perform self-care activities, and inability to perform normal activities	1–5 min	Sagberg et al. [13]	164 patients undergoing surgical resection of a glioma
EORTC QLQ-C30	EORTC-QLQ30 is a modular approach for defining QOL in cancer patients. The original version of this tool was developed for patients participating in international clinical trials	30	physical, role, cognitive, emotional, and social	10–15 min	Mauer et al. [14]	490 patients with glioblastoma
EORTC QLQ-BN20-BCM	Brain tumor-specific module for use with the generalized QOL instrument EORTC QLQ-C30	24	future uncertainty, visual disorder, communication deficits, motor dysfunction, and emotional distress	15–20 min	Osoba et al. [15]	105 patients with brain cancer
PROMIS-10/29	Developed as a subset of the primary PROMIS tool, the PROMIS- 10 and -29 aim to determine the health of patients and their capacity to function over time	10	overall health, quality of life, physical health, mental health, social health, satisfaction with social activities and relationships, independence, pain, fatigue, and emotional health	10–15 min	Gabel et al. [16]	79 patients with glioma
Neuro-QOL	Neuro-QOL is a modular approach to defining QOL in patients with neurological conditions. Researchers select from a question bank and may administer the survey through a computerized adaptive test (CAT)	15–30	Physical, mental, and social health (further categorized into subdomains, including anxiety, depression, fatigue, activities of daily living, lower-extremity function–mobility, cognition–general concerns, cognition–executive function, emotional and behavioral control, overall well-being, sleep quality, ability to participate in social roles and activities, satisfaction with social roles, and sexual function and stigma)	15 min	Rogers et al. [4]	248 long-term survivors with primary CNS tumors
SF-12/SF-36/RAND-36	SF-12 is a short-form version of SF-36, a tool used to measure patient healthcare utilization, mental health, and physical health	12 or 36	physical pain, energy, fatigue, general health perceptions, general mental health, and limitations in physical, social, and usual role activities	10–15 min	Bunevicius et al. [17]	227 patients with brain tumors
LASA	LASA is a self-reported measure of patient QOL, relying on 5 items individual items: physical, spiritual, intellectual, emotional, and overall well-being	5	physical, spiritual, intellectual, emotional, and overall well-being	1–5 min	Coates et al. [18]	205 patients with high-grade glioma
FACT-Br	FACT-BR is a brain tumor-specific version of FACT-G. Includes symptom- and treatment-specific questions for brain tumor patients	27	physical, functional, social, and emotional well-being, and satisfaction with treatment	10–15 min	Roa et al. [19]	100 patients with glioblastoma
MDASI-BT	MDASI-BT is a brain tumor-specific version of MDASI, a general symptom assessment tool for cancer patients	22	pain, fatigue, nausea, disturbed sleep, distress, shortness of breath, difficulty remembering, lack of appetite, drowsiness, dry mouth, sadness, vomiting, numbess and tingling, unilateral body weakness, changes in appearance, changes in bowel pattern, vision problems, seizures, irritability, and difficulty with understanding, speaking, or concentrating	10 min	Piil et al. [20]	120 patients with brain tumors

**Table 2:** Use of ClinRO tools in glioma patients.

Instrument Name	Description	Number of Items	Domains	Time to Complete	Study Characterizing Use	Study Cohort
KPS	Karnofsky Performance Status (KPS) is a clinician-rated scale that assesses a patient’s functional status and ability to perform daily activities.	N/A	N/A	1–2 min	Mackworth et al. [69]	200 brain tumor patients
Neurologic Assessment in Neuro-Oncology (NANO)	The Neurologic Assessment in Neuro-Oncology (NANO) is a standardized clinician-reported outcome measure specifically designed to assess neurological function in neuro-oncology patients through an objective evaluation of nine neurological domains, including gait, strength, ataxia, facial strength, language, visual fields, level of consciousness, behavior, and sensation.	N/A	9	5–10 min	Ung et al. [70]	78 glioma patients

**Table 3:** Use of PerfO tools in glioma patients.

Instrument Name	Description	Number of Items	Domains	Time to Complete	Study Characterizing Use	Study Cohort
Montreal Cognitive Assessment (MoCA)	The MoCA is a tool designed to detect mild cognitive impairment by assessing multiple cognitive domains, including executive function, visuospatial abilities, attention, language, memory, and orientation.	N/A	6	10–15 min	Tymowski et al. [78]	21 patients with low-grade glioma
Trail Making Test (TMT)	The TMT measures visual attention, processing speed, and cognitive flexibility by having patients connect numbered dots (Part A) or alternating numbers and letters (Part B) in sequence as quickly as possible.	N/A	N/A	5–10 min	Smrdl et al. [79]	275 patients with high-grade glioma
Hopkins Verbal Learning Test (HVLT)	The HVLT is a brief verbal learning and memory assessment that evaluates immediate recall, delayed recall, and recognition memory through repeated trials of a 12-word list.	N/A	N/A	15–20 min	Noll et al. [80]	84 patients with temporal lobe glioma
Mini-Mental State Examination (MMSE)	Mini-Mental State Examination (MMSE) is a clinician-administered cognitive screening tool that assesses orientation, attention, memory, language, and visuospatial abilities to identify cognitive impairment.	N/A	5	5–10 min	Brown et al. [57]	203 patients with low-grade glioma

## Data Availability

No new data were created or analyzed in this study. Data sharing is not applicable to this article.
